# “Sounds like CSI:” How consumers of adolescent substance use disorder treatment perceive the term “evidence-based practice”

**DOI:** 10.1186/1940-0640-10-S1-A3

**Published:** 2015-02-20

**Authors:** Sara J Becker, Miriam Midoun, Anthony Spirito

**Affiliations:** 1 Department of Behavioral and Social Sciences, Center for Alcohol and Addictions Studies at Brown University, Providence, RI, 02903, USA; 2 Department of Psychiatry and Human Behavior, Warren Alpert Medical School at Brown University, Providence, RI, 02903, USA

## Background

Recent attempts to increase the utilization of evidence-based practice (EBP) by adolescents with substance use disorders (ASUDs) have included direct-to-consumer marketing and educational materials [1]. For instance, both the National Institute on Drug Abuse and the American Psychological Association’s Division of Child and Adolescent Psychology have developed educational websites for parents of ASUDs that encourage them to seek out those treatments designated as EBP. These educational materials are based on the assumption that ASUDs and their parents will understand the concept of EBP and view the concept favorably. This study aimed to test this assumption and explore how ASUDs and their parents perceive, understand, and react to the concept of EBP.

## Materials and methods

Qualitative focus groups and individual interviews with 29 parents and 24 ASUDs were conducted across a range of treatment settings. Discussions explored four themes: a) familiarity with the concept of EBP; b) assumptions about what EBP means; c) impressions of EBP after reading a common definition; and d) recommended terms to describe EBP in educational materials. Participants’ responses were transcribed verbatim and qualitatively analyzed by two independent coders.

## Results

Only two of the 53 participants had ever heard the term EBP and only one was able to define it correctly. Common assumptions about the term “evidence-based” were that it referred to treatment based on the adolescent’s medical history, legal evidence of the adolescent’s substance use, or the clinician’s prior experience. After reading a common definition of EBP used by national organizations, many participants expressed concerns that the approach sounded inflexible. Multiple participants said that they would prefer a therapy approach described as “individual” or “varied” over an approach described as EBP. Terms the participants recommended to educate potential treatment consumers about EBP included “proven,” “successful,” “better,” and “therapy that works.”

## Discussion

Results of this study indicated that consumers of ASUD treatment had low familiarity with the concept of EBP, incorrect assumptions about what it meant, and negative impressions of the concept even after reading a common definition. Key clinical implications of this study are that attempts to educate treatment consumers about effective treatment should use the phrase “evidence-based” with caution and emphasize the flexibility of the approach.
